# Incidental Hamartoma in an elderly patient: a case report

**DOI:** 10.1186/s12886-020-01604-9

**Published:** 2020-08-17

**Authors:** Tae-Sung Joo, Hyejee Kim, In-Ki Park, Jae-Ho Shin

**Affiliations:** 1 Department of Ophthalmology, Kyung Hee University Hospital, Kyung Hee University School of Medicine, 892, Dongnam-ro, Gangdong-gu, Seoul, Republic of Korea 05278; 2 Department of Medicine, Graduate School, Kyung Hee University, 23, Kyungheedae-ro, Dongdaemun-gu, Seoul, Republic of Korea 02447; 3 Department of Ophthalmology, Kyung Hee University Hospital, Kyung Hee, University School of Medicine, 23, Kyungheedae-ro, Dongdaemun-gu, Seoul, Republic of Korea 02447

**Keywords:** Adult neuromuscular Hamartoma, Hypertropia, Incidental orbital mass, Inferior rectus muscle palsy

## Abstract

**Background:**

Neuromuscular hamartoma is a very rare tumor; with only five cases reported in the orbit. It often occurs in infants and young children and involves large peripheral nerves, but there has been only few reports of occurrence in the orbit and adults.

**Case presentation:**

This paper describes a 70-year-old man with an incidental orbital mass detected by an imaging test and who later developed associated symptoms. The mass was diagnosed as neuromuscular hamartoma. Superior rectus muscle recession and inferior rectus muscle resection were performed in the right eye for hypertropia secondary to postoperative inferior rectus muscle palsy. Hypertropia in the right eye and diplopia improved after surgery, and regular follow-up is underway.

**Conclusion:**

This is the first case of an incidentally detected orbital mass diagnosed by histologic examination as a neuromuscular hamartoma in an older patient whose proptosis progressed after a long period of inactivity.

## Background

Neuromuscular hamartoma, also known as neuromuscular choristoma or benign triton tumor, is a rare benign tumor with well-differentiated, mature, striated muscle and nerve fibers [1]. In most cases, it involves large nerves, such as the brachial plexus or sciatic nerve [2], and typically appears in infants and young children. It is not common to find it in both the head and in adults [3].

According to a previously published report, there have been four neuromuscular hamartomas reported in adults and in the orbit [1, 3–5]. Therefore, we report a case of an orbital mass diagnosed as a neuromuscular hamartoma in an older male patient with proptosis.

Informed written consent was obtained from the patient for publication of this case report and accompanying images.

## Case presentation

**Fig. 1:**
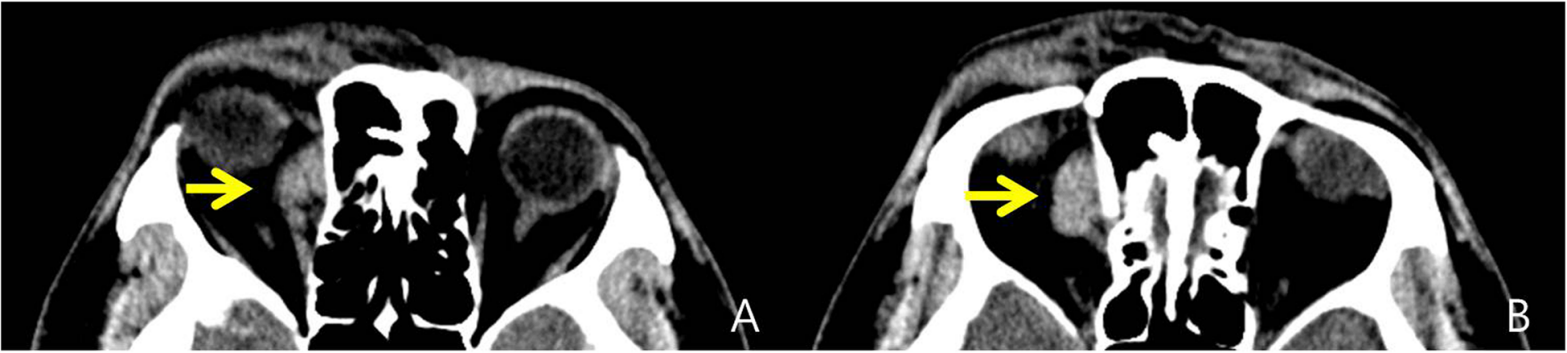
Axial computed tomography scans show a homogenous well-enhanced mass (2.4 × 1.5 cm) at the superomedial aspect of the right orbit

A 70-year-old man presented to the emergency department with sudden-onset chest discomfort and dizziness. Computed tomography (CT) scan revealed encephalomalacia in the right ventral frontal lobe with a mass in the superomedial aspect of the right orbit (Fig. 1). However, he had no ophthalmologic symptoms, and no specific treatment was administered for the mass.

**Fig. 2:**
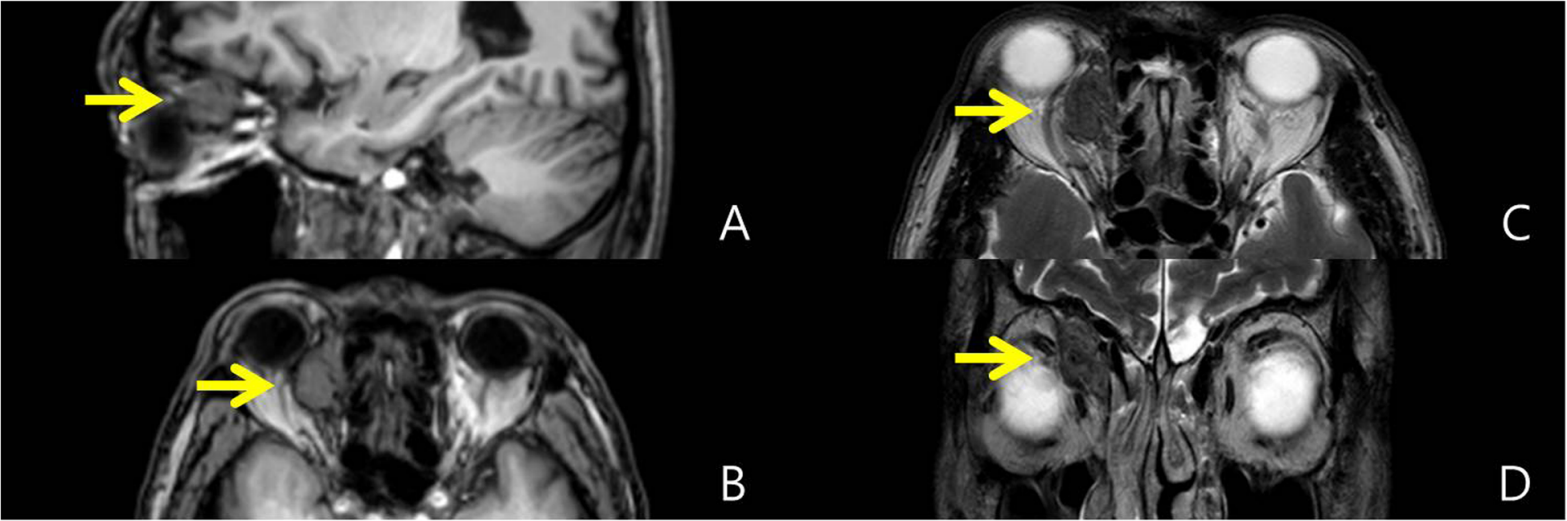
**a** T1-sagittal and **b** T1-axial, **c** T2-axial, and **d** T2-coronal magnetic resonance images reveal a solid mass (2.4 × 1.5 × 2.0 cm) at the superomedial aspect of the right orbit with T1 and T2 iso-signal intensity. There was no interval change in the mass compared with a previous CT

Five years later, the patient presented to the neurology clinic complaining of headache. Magnetic resonance image (MRI) showed a solid mass (2.4 × 1.5 × 2.0 cm) at the superomedial aspect of the right orbit with T1 and T2 iso-signal intensity (Fig. 2). Comparison with a previous CT (Fig. 1) showed no change in the mass.

**Fig. 3:**
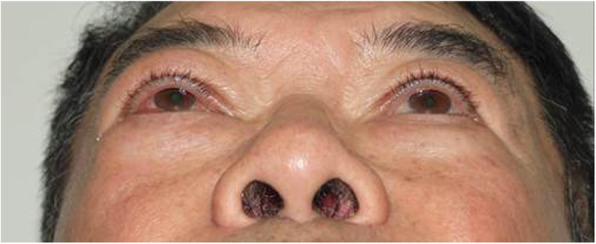
Photograph of proptosis of the patient’s right eye. The proptosis was 5 mm

**Fig. 4:**
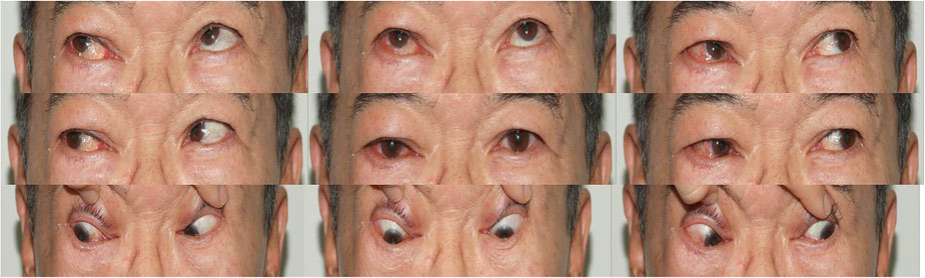
A 9-cardinal photograph showed a subconjunctival hemorrhage, chemosis with proptosis of the right eye, and downward ocular deviation of the right eye, but the EOM was normal

One month later, the patient presented to the ophthalmology clinic with right eye proptosis. On ophthalmologic examination, the degree of proptosis was 3 mm, but external ocular movement (EOM) was normal. A conservative management approach was decided based on the assumption of a benign mass. However, 2 years later, proptosis of the right eye had increased to 5 mm (Fig. 3), and signs of subconjunctival hemorrhage, chemosis, and downward ocular deviation of the right eye were noted in 9-cardinal photograph, but the EOM was normal (Fig. 4).

**Fig. 5:**
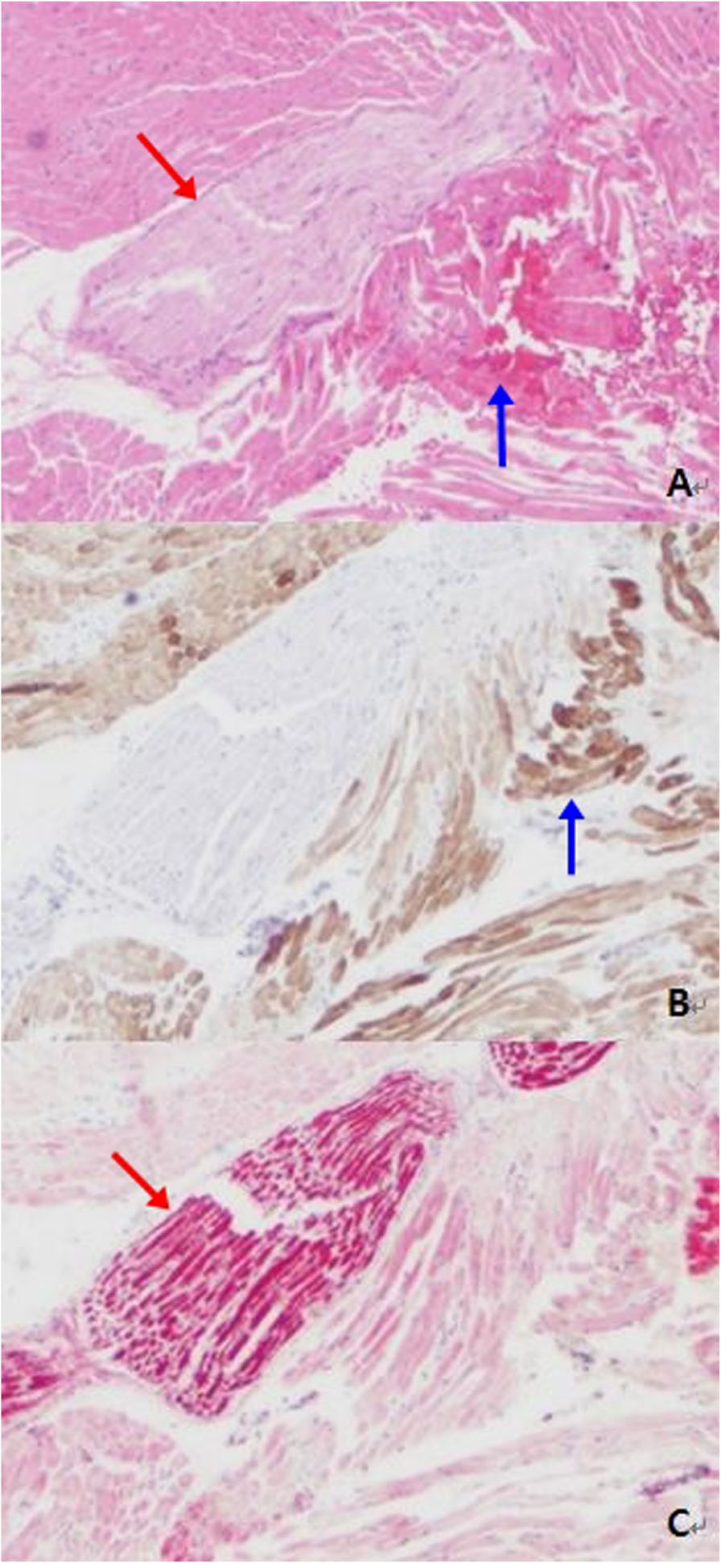
Photomicrographs of the resected lesion. **a** Hematoxylin & eosin (H&E) staining shows mature muscular tissue (blue arrow) admixed with proliferation of nerve tissue (red arrow) (× 40). **b** Immunohistochemical staining for Desmin was positive in muscle tissue (blue arrow). (× 40) **c** Immunohistochemical staining for S-100 was positive in nerve tissue (red arrow) (× 40)

One month later, an excisional biopsy was performed through a medial canthal incision to almost completely excise the brown-colored tissue. After incision, the thin capsulated mass was easily found and there was no adhesion with the surrounding tissue or rectus muscle, although it was difficult to remove completely due to bleeding. A histopathological evaluation of the tissue revealed a neuromuscular hamartoma with mature muscular tissue and proliferation of nerve tissue; the specimen was Desmin positive in muscle and S-100 positive in nerve tissue (Fig. 5).

**Fig. 6:**
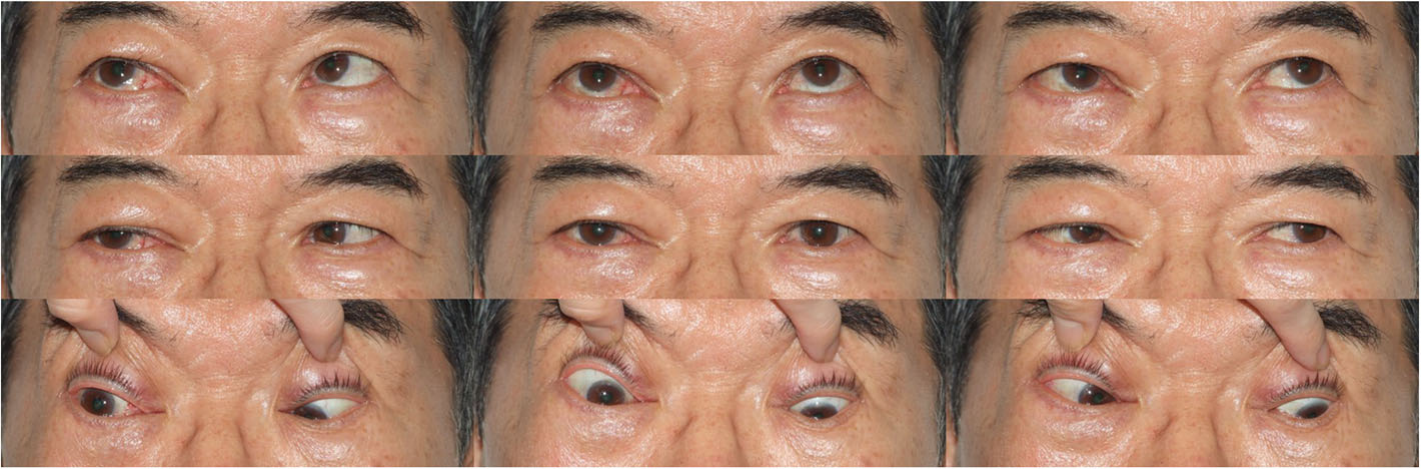
A 9-cardianl photograph showed abnormal ocular movement. In particular, downward gaze limitation was observed

Postoperative steroid tapering was administered. At a follow-up visit on postoperative day five, the patient complained of vertical diplopia. Conservative management was applied because it was thought to be caused by conjunctival swelling over the inferior rectus muscle-side conjunctiva. Although exophthalmos and conjunctival swelling decreased, diplopia has worsened with EOM increased to 15 prism RHT and downward gaze limitation. Therefore, inferior rectus (IR) muscle resection (2 mm) & superior rectus (SR) muscle recession (3.5 mm) were performed based on diagnosis of inferior rectus muscle palsy (Fig. 6).

Diplopia persisted after strabismus surgery, for which a prism glasses prescription was administered to relieve the symptom. Nearly, one year after strabismus surgery, The condition improved to around 4 to 6 prism RHT.

## DISCUSSION and CONCLUSION

Finding neuromuscular hamartoma in the orbit is unusual; there have been only five reported cases [1, 3–6]. After a review of the literature (Table. 1), we believe this is the first case of this tumor occurring in an adult patient with age of 65 years and older [7–13] as it is known to mostly occur in infants and children [12, 13]. The proximal aspects of large peripheral nerves such as the brachial and sciatic nerves are most commonly involved with only a few cases involving the head and neck regions [10, 11, 13, 14]. One report found that this tumor is more likely to occur in women than men, with a 2.4 to 1 ratio [11].

**Table 1:** A literature review of neuromuscular hamartoma cases in the orbit

Author	Age	Gender	Location by radiological examination (MRI or CT)
Iferkhass et al. (2015)	47	F	Infero-lateral portion of right orbit
Bae et al. (2014)	53	F	Supero-medial portion of right orbit (surrounding optic nerve and ocular muscles in the right retrobulbar area)
Cunniffe et al. (2010)	61	M	Left superior rectus muscle and overlying soft tissue
Perry et al. (2017)	53	M	Left orbital apex abutting the inferior aspect of the optic nerve
Oeppen et al. (2003)	2.5	M	Inferior orbital fissure and lateral wall of the orbit and filled the posterior part of the orbit

Neuromuscular hamartomas typically show moderate patchy enhancement with low or intermediate signal intensity on MRI [6] and are hyperattenuated on CT [9]. Lymphadenopathy is not seen. Simple moderate enhancement is not compatible with rhabdomyosarcoma, lymphoma, and nasopharyngeal carcinomas [15]. Because imaging features are not characteristic, neuromuscular hamartoma must be considered in the differential diagnosis of an orbital mass in a young child.

According to Daley et al. [10], neuromuscular hamartoma was divided based on the site of origin into two groups: an aggressive central type and a non-aggressive peripheral type. The central type invaded large intracranial nerves or infratemporal fossa nerve trunks, occasionally causing muscle atrophy and weakening. It most commonly extended to the infratemporal fossa via the foramen ovale by invading the fifth cranial nerve in or near the middle cranial fossa (specifically Meckel’s cave) [6, 9, 12, 13, 16]. These cases occurred in infants and children and required surgical treatment. The peripheral type was found in subcutaneous or submucosal tissues as non-encapsulated lumps and was asymptomatic, non-destructive, and slowly increased in size. This group of tumor was found in patients with older age, including adulthood [11, 14]. Surgical resection was simple and curable. Of the nine cases, five were central type, and four were peripheral type.

Clinical symptoms of neuromuscular hamartoma vary from asymptomatic to pain or neurologic deficit. Most tumors have no symptoms, but pain and neurological dysfunction such as paresthesia and muscle weakness, ophthalmoplegia, and migraine may occur depending on location [9, 12, 17].

Pathologically, the specimens were stained with hematoxylin and eosin and trichrome. Small nerve bundles and axons were seen intermixed with adjacent mature striated skeletal muscle bundles, surrounded by a dense collagenous matrix. The skeletal muscle noted to have small, bland, peripherally-placed nuclei with mature features and no cellular atypia or atypical mitoses. Immunohistochemically, S-100 and neurofilament stains showed normal nerve bundles. Desmin highlighted the hamartomatous striated muscle fibers intermingled with nerve bundles [18].

Treatment for neuromuscular hamartoma is excision for the aggressive central type and conservative management for the peripheral type. Incomplete excision can alleviate symptoms. Although most tumors have a good prognosis after resection, recurrence has been reported [11]. Therefore, physicians must closely follow patients after treatment. It is also important to preserve neural function during treatment, because nerve palsy may occur as a complication and is irreversible [8, 10, 19].

In the present case, the patient suffered from diplopia secondary to postoperative hypertropia in the affected eye. Although IR muscle resection & SR muscle recession were performed for correction, diplopia persisted for two months after strabismus surgery. It is thought that the inferior rectus muscle was paralyzed due to either compression by a retractor used to obtain a surgical field of view and control bleeding or direct damage during the operation. However, it is difficult to explain the precise mechanism as the tumor site was in the superomedial region.

The present case describes an orbital mass in the right eye of an elderly patient that was incidentally found in a head and neck imaging study. During follow up, proptosis progressed; thus surgical resection and biopsy were performed. Histological examination confirmed the mass as a neuromuscular hamartoma. This is the first case of an incidentally-discovered orbital mass that was diagnosed as neuromuscular hamartoma in an elderly patient whose proptosis progressed after a long period of inactivity.

## Data Availability

The datasets from the current study can be obtained on request from the corresponding author.

## Abbreviations

EOM: External ocular movement
RHT: Right hypertropia
IR: Inferior rectus
SR: Superior rectus
H&E: Hematoxylin & eosin
